# Temporal dynamics of SARS-CoV-2 detection in wastewater and population infection trends in Mexico City

**DOI:** 10.3389/fpubh.2025.1640581

**Published:** 2025-08-14

**Authors:** Miguel Atl Silva-Magaña, Marisa Mazari-Hiriart, Adalberto Noyola, Ana C. Espinosa-García, Guillermo de Anda-Jáuregui, Enrique Hernández-Lemus

**Affiliations:** ^1^Computational Genomics Division, National Institute of Genomic Medicine, Mexico City, Mexico; ^2^Laboratorio Nacional de Ciencias de la Sostenibilidad, Instituto de Ecología, Universidad Nacional Autónoma de México, Mexico City, Mexico; ^3^Instituto de Ingeniería, Universidad Nacional Autónoma de México, Mexico City, Mexico; ^4^Investigadores por México, National Council for Science and Technology, Mexico City, Mexico

**Keywords:** waste-water based epidemiology, COVID-19, SARS-CoV2, temporal dynamics, predictive models, early signals

## Abstract

Wastewater-based epidemiology (WBE) provides a non-invasive, community-level approach to monitor infectious diseases such as COVID-19. This study investigated the temporal relationship between SARS-CoV-2 RNA levels in wastewater and reported COVID-19 cases in adjacent populations in Mexico City. A total of 40 samples were collected from the Copilco neighborhood during two epidemiological waves (April-September 2021 and November 2021-February 2022). An optimized one-step RT-qPCR protocol targeting the N1 gene achieved 96.7% efficiency with a detection limit of 10 copies/μL. Spatial classification identified three proximity zones based on drainage system topology. Cross-correlation analysis between viral genome copies and confirmed case data revealed a significant temporal lag of 6-8 days. These results support the application of WBE as an early-warning tool to inform public health strategies and anticipate infection trends.

## 1 Introduction

Emerging infectious diseases (EIDs), such as COVID-19, represent a persistent global health challenge, characterized by their rapid spread and significant societal impact (1–4). Defined as infections that have newly appeared or increased in incidence within the last two decades, EIDs like SARS-CoV-2 pose a critical burden on health systems worldwide (5–7). These diseases often overwhelm healthcare infrastructure, necessitate substantial investments in treatment and vaccination, and exacerbate pre-existing health disparities (8, 9). In Mexico, the COVID-19 pandemic has highlighted vulnerabilities in healthcare access and response capabilities, particularly in densely populated urban centers (10–16).

The SARS-CoV-2 virus, responsible for COVID-19, spreads primarily via respiratory droplets but has also been detected in fecal matter, suggesting wastewater as a potential surveillance medium. Wastewater-based epidemiology (WBE) has gained traction as a non-invasive tool to monitor viral prevalence within communities (17–21). Unlike traditional epidemiological approaches, WBE provides a cost-effective and comprehensive snapshot of population-level infection dynamics, encompassing symptomatic and asymptomatic cases (22–24). Such methodologies are particularly relevant in low-resource settings where widespread clinical testing may be unfeasible.

Globally, WBE has proven effective in early outbreak detection, guiding public health interventions, and estimating disease prevalence (25, 26). The approach offers distinct advantages for public health and policy. First, it enables real-time monitoring of infection trends at a community scale, reducing the need for costly individual-level testing. Second, WBE can identify asymptomatic carriers who might otherwise go undetected in traditional surveillance systems (27–29). In parallel, WBE has enabled the early identification of emerging variants, often several days or even weeks ahead of clinical sampling, underscoring its potential utility for genomic surveillance (30–33). Finally, it supports proactive health policy decisions by providing data that inform resource allocation, intervention timing, and public communication strategies. These advantages make WBE an invaluable component of integrated public health surveillance systems (34, 35).

In Mexico City, one of the areas most severely impacted by COVID-19, leveraging WBE presents a unique opportunity to strengthen epidemiological surveillance and inform policy decisions (12, 13, 36, 37). This study evaluates the temporal relationship between SARS-CoV-2 RNA levels in wastewater from the Copilco neighborhood and reported infection trends, aiming to validate WBE's role in public health preparedness and response.

## 2 Methods

**Figure 1:**
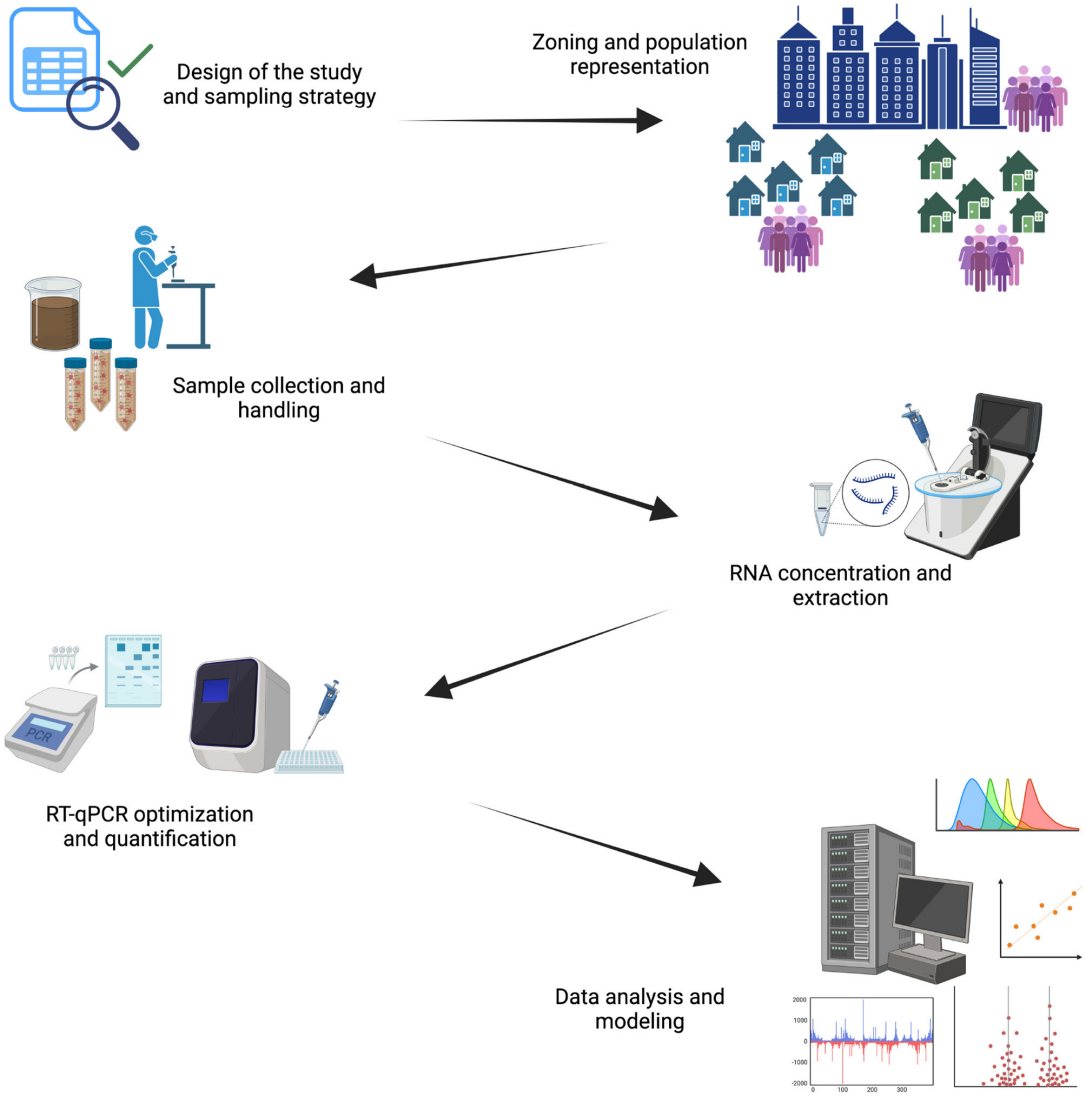
Workflow for this work. Created in https://BioRender.com.

A diagrammatic scheme with the methods described here is presented in the form of a workflow in the Figure 1.

### 2.1 Study design and sampling

This study was conducted in the Copilco neighborhood of Mexico City, a densely populated urban area with a complex drainage network. A total of 40 wastewater samples were collected across two sampling seasons: April to September 2021 (*n* = 18) and November 2021 to February 2022 (*n* = 22). These periods were selected based on local epidemiological data indicating significant COVID-19 case peaks. Sampling was performed at a drainage point located near Copilco Metro station (19.335757 N, -99.176893 E), chosen for its accessibility and strategic position within the local drainage system.

### 2.2 Zoning and population representation

**Figure 2:**
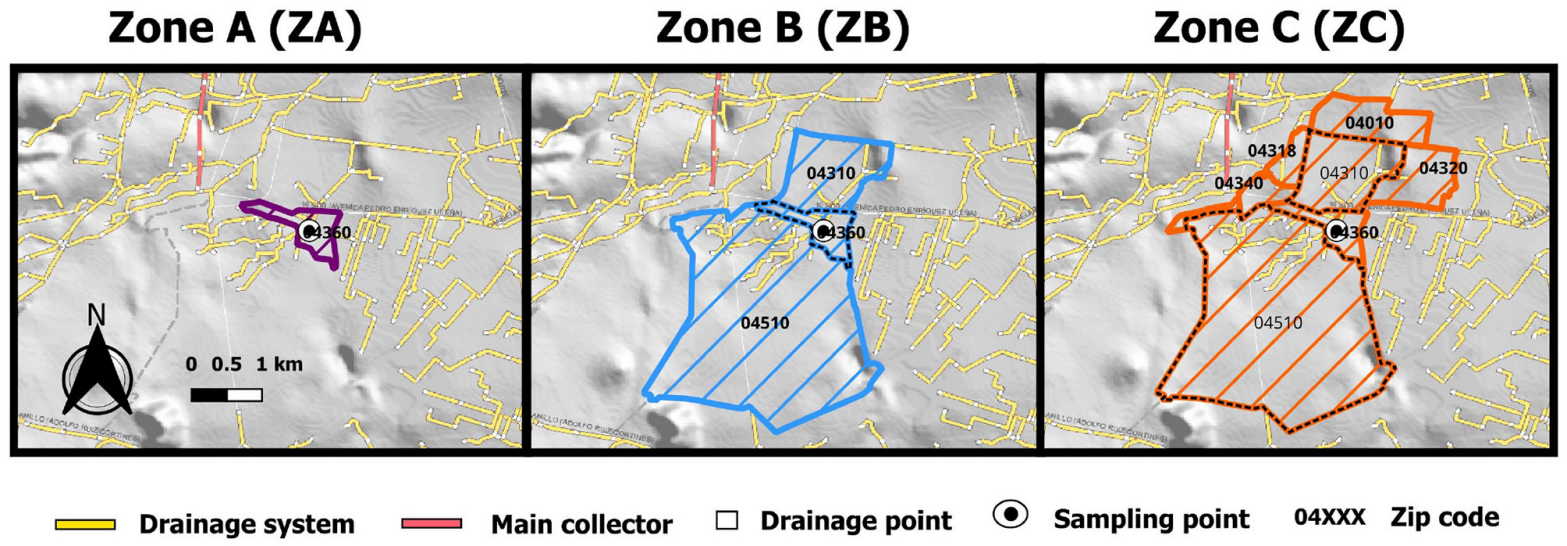
Zones (ZA, ZB, ZC) defined for analysis, based on proximity to the sampling point, using postal codes and the wastewater drainage map.

To assess spatial resolution, the area was divided into three nested analysis zones based on drainage topology, encompassing different distances from the sampling point. Postal codes were used as unique proximity identifiers, as follows: Zone A (ZA) comprised only the postal code where the sampling point was located; Zone B (ZB) included the ZA and the immediately subsequent postal codes connected by a drainage line; and Zone C (ZC) included the ZA-ZB and the immediately subsequent postal codes connected by a drainage line (Figure 2). The reported number of inhabitants for ZA is 8,458, for the ZB it is 22,099, and for ZC it is 43,204. According to the latest Population and Housing Census for Mexico City 2020 (38). Using the postal codes associated with each zone, local reported cases specific to each zone were obtained from the Mexican National Epidemiological Surveillance System (SISVER) database. Spatial mapping and zoning were performed using QGIS software (version 3.24.3) (39).

### 2.3 Sample collection and handling

Samples were collected following the Centers for Disease Control and Prevention (CDC) guidelines for wastewater surveillance (https://www.cdc.gov/nwss/wastewater-surveillance.html) (40), emphasizing safety and contamination prevention. Each sample was taken around 10 a.m., using sterile polypropylene containers (1 L capacity) and transported at 4°C to the laboratory for processing within 24 h.

### 2.4 Concentration and RNA extraction

Wastewater samples were concentrated using a polyethylene glycol (PEG) precipitation method (41, 42), modified for higher recovery efficiency (43). A 200 mL aliquot of each sample was mixed with 25 mL of Tris-Glycine-Extract Broth (TGEB, pH 9.5), agitated for 2 h at 4°C, and centrifuged at 2,500 g for 10 min. The supernatant was adjusted to pH 7.0–7.2 and precipitated using 20% PEG 8000 and 0.3 M NaCl. After overnight incubation at 4°C with agitation, the samples were centrifuged at 10,000 g for 30 min. Pellets were resuspended in 0.5 mL of phosphate-buffered saline (PBS) and stored at -80°C until RNA extraction. RNA was extracted using the QIAGEN QIAamp Viral RNA Mini Kit, yielding a final volume of 60 μL per sample.

### 2.5 RT-qPCR optimization and quantification

The RT-qPCR protocol targeted the N1 gene of SARS-CoV-2, using forward primer 5'-GACCCCAAAATCAGCGAAAT-3', reverse primer 5'-TCTGGTTACTGCCAGTTGAATCTG-3' and probe 5'-FAM-ACCCCGCATTACGTTTGGTGGACC-BHQ1-3' sequences (44). Quantification standard curves were prepared using the synthetic control VR-3276T (ATCC) with a range between 10^1^ and 10^4^ copies of the N1 gene. All samples were analyzed in triplicate, including negative controls (RNase-free water) and parallel detection of rotavirus A nonstructural protein 5 gen (NSP5) was performed using a standard genesig kit from Primerdesign Ltd. as an internal control. Thermal cycling was performed on an Applied Biosystems StepOnePlus system. Key optimizations included: annealing-extension temperature, adjustment of magnesium chloride (MgCl_2_), primer and probe concentrations to improve efficiency, and reduction of reaction volume to 10 μL.

### 2.6 Data analysis

The model considers the three defined zones ZA, ZB, ZC for spatial analysis and three time blocks: TB1: April 2021 to February 2022 (total time represented); TB2: April 2021 to September 2021 (sampling session 1); TB3: November 2021 to February 2022 (sampling session 2) for temporal analysis. Time series for interpolated viral RNA counts and reported COVID-19 cases were smoothed using a 7-day simple moving average (SMA 7) (45–47). Cross-correlation analysis (48–50) was performed to identify temporal lags between SARS-CoV-2 RNA levels and reported COVID-19 cases. The analysis included statistical tests for differences between sampling seasons using the Wilcoxon signed-rank test (51–53). The R statistical programming language (version 4.2.1) using the “ xcorr” function. The cross-correlation estimate is thus calculated by a *spectral* method in which the Fast Fourier Transform (FFT) of the first vector is multiplied element-by-element with the FFT of second vector. The computational burden of this algorithm depends on the length N of the vectors and is independent of the number of lags. Wilcoxon signed-rank test were calculated using the “ wilcoxon.test” function of the “MASS” R-library (54, 55).

## 3 Results

### 3.1 RT-qPCR optimization

**Figure 3:**
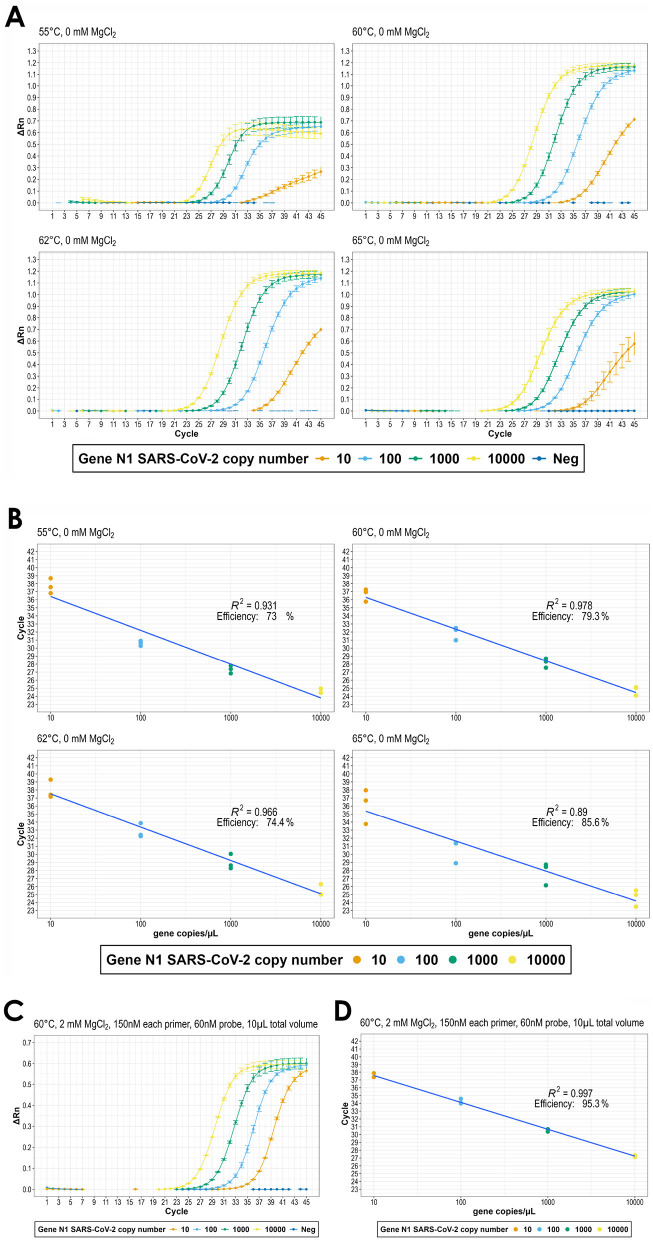
Outline of the RT-qPCR analytical method. **(A)** amplification panel ( Δ*R*_*n*_ vs. Cycle) and **(B)** associated regression panel (copies/μL N1 gene vs. Cycle), for annealing-extension temperatures 55°C, 60°C, 62°C, 65°C and 25 μL final volume; **(C)** amplification (Δ*Rn* vs. Cycle) and **(D)** associated regression (copies/μL N1 gene vs. Cycle) for optimized RT-qPCR (60°C, MgCl2 (2 mM), forward and reverse primers (150 nM), probe (60 nM) and 10 μL final volume.

The RT-qPCR protocol was successfully optimized to achieve high sensitivity and efficiency. By reducing reaction volumes to 10 μL and fine-tuning magnesium chloride concentrations, oligonucleotide levels, and probe quantities, an efficiency of 96.7% was achieved with a detection limit of 10 copies/μL. The optimized conditions reduced reagent usage while maintaining robust performance, offering a cost-effective alternative to commercial kits. Final reaction conditions were as follows: 50°C for 5 min 95°C for 20 s (enzyme activation), 45 cycles of 95°C for 5 s and 60°C for 20 s. The associated results of the initial and final optimized conditions can be seen in Figure 3.

1. 2.5 μL of 4X Master Mix (Applied Biosystems, A28525)
2. 1 μL each of forward and reverse primers (150 nM)
3. 1 μL of probe (60 nM)
4. 0.4 μL of MgCl2 (2 mM)
5. 3 μL of RNA template
6. 1.1 μL of nuclease-free water

### 3.2 Quantification of viral RNA

**Figure 4:**
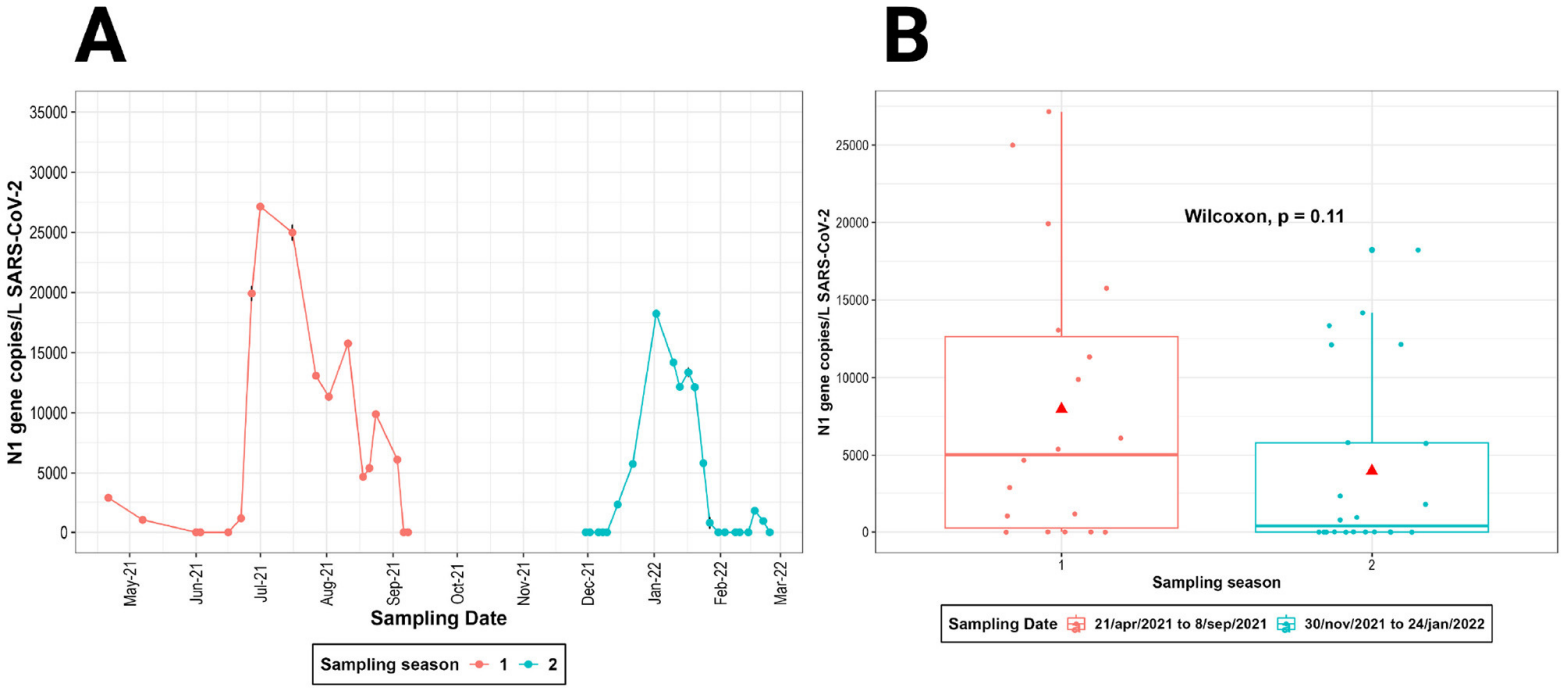
SARS-CoV-2 copies measured on each sampling dates. **(A)** Sampling dates for season 1 and season 2. **(B)** Boxplots of SARS-CoV-2 copies showed no statistical differences in the two sampling seasons.

N1 gene copy number counts per liter (copies/L) in wastewater samples ranged from 10^3^ to 10^5^. Temporal trends revealed distinct peaks in viral RNA levels during the sampling seasons, corresponding to reported COVID-19 case surges. The first season (April–September 2021) showed a gradual increase, peaking in July 2021, while the second season (November 2021–February 2022) exhibited sharper spikes in December 2021 (Figure 4).

### 3.3 Spatial analysis of infection trends

**Figure 5:**
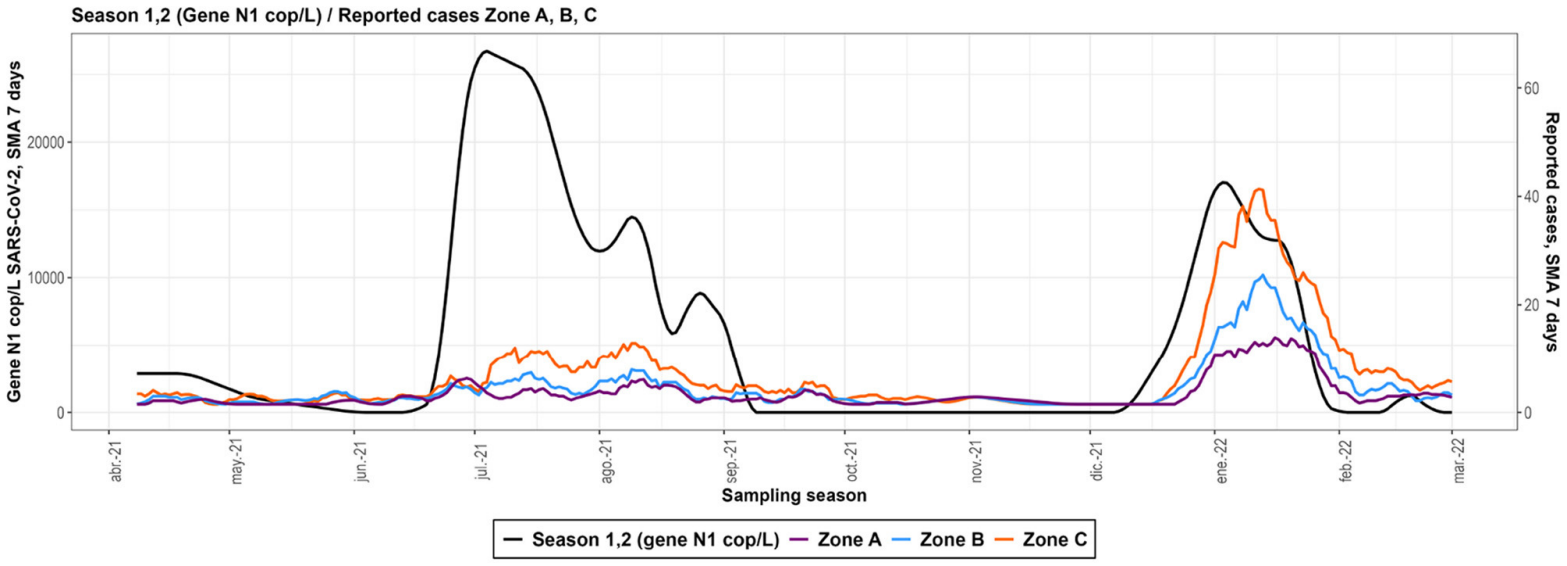
Smoothed curves depicting the full sampling season. Black curve with scale on the left y-axis is the measured concentration of SARS-CoV-2 whereas red blue and green curves with scale on the right y-axis are the number of cases in zones **A**, **B**, and **C** respectively.

**Figure 6:**
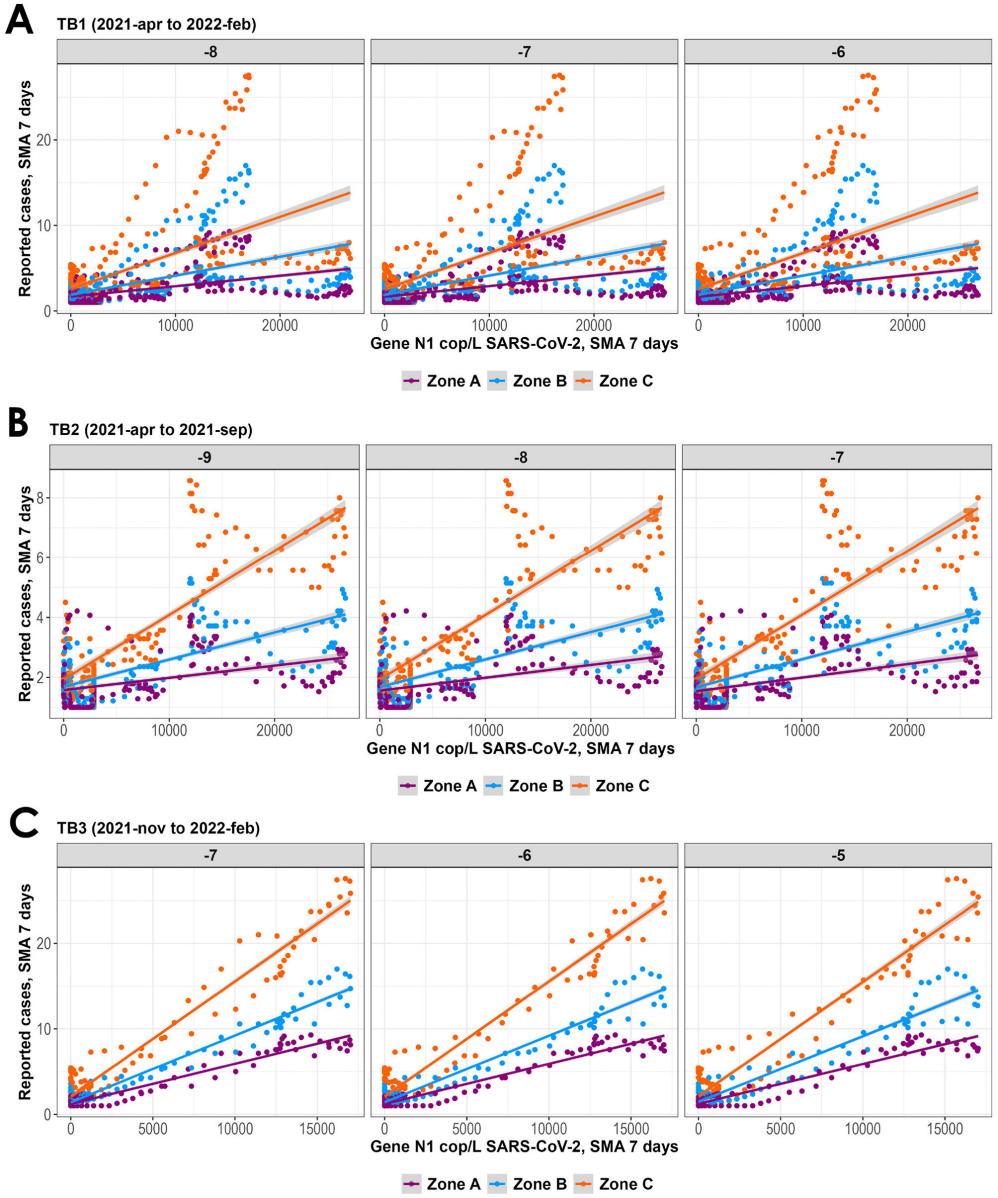
Scatter plot behavior for the different defined zones: ZA,ZB and ZC across selected time lags (only the most relevant are shown) and date ranges. **(A)** TB1 April 2021 to February 2022; **(B)** TB2 April 2021 to September 2021; **(C)** TB3 November 2021 to February 2022.

**Figure 7:**
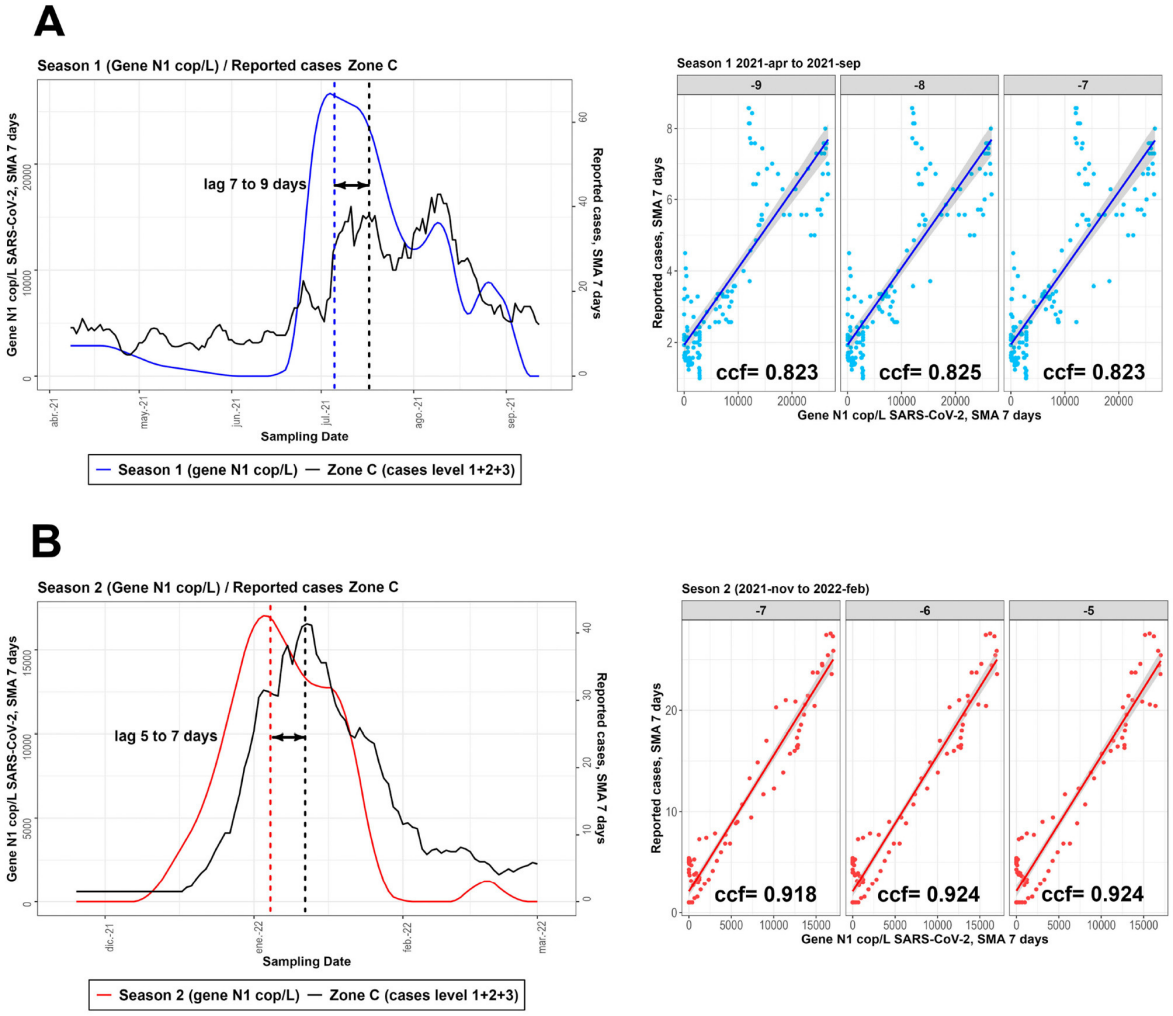
Graphs and cross-correlation results between the concentration of measured viral copies and reported cases for season 1 **(A)** and season 2 **(B)** for the analysis of Zone C.

The analysis of the relationship between SARS-CoV-2 viral load in wastewater and clinically reported cases in three geographical areas studied (ZA, ZB and ZC) revealed a positive correlation, more pronounced in Zone C, which covers a wider area (Figures 5, 6). This trend was consistent across the different temporal periods analyzed, becoming more evident during period TB3, corresponding to the sampling season 2 (Figure 7). The steeper slope observed in the data from Zone C suggests that spatial integration over a larger geographic scale allows for a more robust identification of the relationship between viral circulation and reported cases. However, this approach requires careful consideration of the associated population size, as expanding the spatial coverage may also increase data variability and, consequently, weaken the strength of the observed association between the variables.

### 3.4 Temporal correlation analysis

Cross-correlation analysis revealed significant time lags between viral counts in wastewater and reported infections. In TB1, the highest correlation (ccf = 0.571) was observed with a 7-day lag; in TB2, the strongest correlation (ccf = 0.825) was observed with an 8-day lag; and in TB3, the highest correlation (ccf = 0.924) was observed with a 6-day lag. These results can be seen in Table 1 and the associated graphs in the Supplementary material (Supplementary Figure S1). The cross-correlation values obtained throughout the process can be compared in the Supplementary material (Supplementary Figure S2 and Supplementary Table S1), which contain the values obtained using only the interpolation process and using interpolation and smoothing. Figure 7 shows the results for the ZC, which showed the highest correlation values. These results in particular show similar trends to those found in other studies (56–58) where it was possible to find maximum infection peaks in advance using WBE of SARS-CoV-2 with a variable time advantage.

**Table 1:** Results of the cross-correlation factor (CCF) analysis for the combinations between the defined study zones (ZA, ZB, ZC) and the different time blocks (TB1, TB2, TB3).

	**TB1**		**TB2**		**TB3**	
	**lag (days)**	**ccf**	**lag (days)**	**ccf**	**lag (days)**	**ccf**
**Zone A**	4D	0.498	-3D	0.678	6D	0.922
	3D	0.498	-2D	0.676	5D	0.919
	5D	0.496	-4D	0.672	7D	0.917
	2D	0.494	-1D	0.669	4D	0.909
	6D	0.492	-5D	0.662	8D	0.905
	1D	0.486	0D	0.657	3D	0.892
	7D	0.485	-6D	0.649	9D	0.887
	8D	0.477	-7D	0.634	2D	0.866
	0D	0.475	1D	0.631	10D	0.863
	9D	0.467	-8D	0.619	11D	0.834
**Zone B**	7D	0.530	0D	0.733	6D	0.924
	6D	0.529	1D	0.730	7D	0.923
	8D	0.527	2D	0.725	5D	0.918
	5D	0.526	-1D	0.723	8D	0.916
	9D	0.521	3D	0.719	4D	0.906
	4D	0.520	4D	0.714	9D	0.901
	10D	0.512	-2D	0.710	3D	0.887
	3D	0.512	5D	0.709	10D	0.879
	2D	0.501	6D	0.703	2D	0.864
	11D	0.501	-3D	0.696	11D	0.851
**Zone C**	7D	0.571	8D	0.825	6D	0.924
	6D	0.570	7D	0.823	5D	0.924
	8D	0.569	9D	0.823	7D	0.918
	5D	0.566	6D	0.819	4D	0.917
	9D	0.563	10D	0.818	8D	0.904
	4D	0.559	5D	0.813	3D	0.903
	10D	0.554	11D	0.810	9D	0.884
	3D	0.549	4D	0.805	2D	0.883
	11D	0.542	12D	0.798	1D	0.857
	2D	0.537	3D	0.796	10D	0.856

## 4 Discussion

This study reinforces the value of wastewater monitoring as a surveillance tool, particularly in urban settings. The significant time lags observed indicate that wastewater monitoring can provide early warnings of infection spikes, enabling proactive public health responses. While this study focused on a single sampling site, the surrounding population ranges from 8,000 to 43,000 inhabitants, depending on the zone (A, B, C) analyzed. This is a strategic location, as it serves as a wastewater collection point for the surrounding neighborhoods. Due to these characteristics, the study assumes high local representativeness, but expanding the sampling network to increase coverage and reliability could improve the representativeness of Mexico City.

It should be noted that a formal power analysis was not performed due to the lack of standardized methods for estimating power in time-lagged WBE studies of SARS-CoV-2 viral RNA concentrations at the time of sampling. This work should be considered an exploratory analysis demonstrating the feasibility of detecting temporal correlations between SARS-CoV-2 N1 gene quantifications in wastewater and reported infections in a specific urban context. Future work would benefit from larger sample sizes and power calculations based on the effect sizes and time lags observed here. Furthermore, the detection of SARS-CoV-2 RNA in wastewater only reflects the presence of genomic material and does not guarantee viral viability (59–61). Therefore, the observed peaks should be interpreted as a population indicator of epidemiological trends useful for monitoring but not as a direct measure of the risk of community transmission.

To address missing data from dates without sample collection or reported cases, data interpolation was performed for both datasets. A higher number of interpolated values were required for wastewater samples, though an expected trend of genome count fluctuations was observed. Additionally, data smoothing was applied to reduce noise and facilitate subsequent analysis, ensuring the identification of generalizable patterns within the model's variable constraints.

To conduct an exploratory graphical analysis of the potential temporal relationship between both datasets, a scatter plot analysis was performed. This involved shifting the genome count data by one-day increments relative to the number of reported cases, within a time-lag window ranging from 14 days before to 5 days after. The analysis was carried out using the complete dataset from both sampling periods combined (TB1) and separately for each sampling period (TB2 and TB3). As it was previously mentioned, a cross-correlation analysis was performed. In all cases, the cross-correlation factor (CCF) is higher for TB2 and TB3, that is, when the sampling seasons are analyzed separately. It is also greater for Zone C, which corresponds to the sum of infection cases reported in Proximity Levels 1, 2, and 3. Additionally, the time lags with the highest CCF values correspond to the scatter plots with the steepest slopes. This suggests that the relationship improves when analyzing time periods with better representativity of sampled days and a higher number of reported cases. Therefore, these two parameters were continuously refined to enhance the proposed model.

For TB2 (April 2021 to September 2021), the highest CCF (0.823 to 0.825) corresponds to a lag of 7 to 9 days before the reported cases in the population. In contrast, for TB3 (November 2021 to February 2022), the highest CCF (0.918 to 0.924) corresponds to a lag of 5 to 7 days before the reported cases. As it was shown in the Results section, these time windows align with the steepest slopes in the previous exploratory graphical analysis, indicating the period before detection through direct epidemiological evaluation in the population.

One of the first studies conducted to evaluate the correlation between the presence of SARS-CoV-2 genomes in wastewater and reported cases in the population analyzed data from treatment plants in six cities and an airport in the Netherlands. It found the presence of viral particles 7 to 9 days in advance using RT-qPCR (56). Another study evaluating 32 treatment plants in Catalonia, Spain, detected viral genomes 7 days in advance. It also assessed different population sizes and models, concluding that understanding population dynamics can lead to a more accurate predictive model (57). In a separate study that collected a total of 1,101 samples from various treatment plants and sewer systems in Paris, France, it was found that SARS-CoV-2 genomes could be detected 3 to 4 days in advance, particularly in populations with limited mobility. The variation in detection also depended significantly on the time of sample collection mainly due to the different dynamics of population behavior (62, 63).

A more recent study conducted in Xàtiva, in the province of Valencia, reported predictive windows of 15 to 17 days using different models and hospitalization data. This was facilitated by a more established local sampling strategy and a well-documented population dynamic (64). Meanwhile, another study in Yamanashi Prefecture, Japan, reported a predictive window ranging from 3 to 9 days (58).

It is important to note that some longitudinal studies indicate that a proportion of those infected shed SARS-CoV-2 RNA in feces days before the onset of symptoms, but shedding can also last several weeks, so the exact magnitude of this presymptomatic phase varies between populations and viral lineages (65–67). Therefore, inference of time lags from WBE should be interpreted with caution due to this uncertainty, and the set-up of a monitoring system should consider these issues (61, 68).

A key aspect of this study was the selection of populations associated with the sampling points. This was done by integrating information on drainage systems with population sizes linked to postal codes, defining an area of influence around the selected collection point. This approach allowed for the delineation of reported infection data corresponding to the sampling site. This is particularly important because infection dynamics may differ in other areas, and including additional populations could increase variability in the model, making it less robust.

All these findings indicate that predictive time windows can vary across different locations depending on the population and site context. The inclusion of intrinsic variables related to the population's dynamics, along with a detailed understanding of wastewater characteristics and additional health system data, could help refine these predictive models. In the case of this study, the determination of the geographic area and, consequently, the study population was appropriate; however, incorporating a parameter to normalize the size population size associated with each sample could help reduce result variability if applied correctly. Therefore, it is recommended to explore measurement strategies such as genes associated with *Pepper mild mottle virus* (PMMoV) or physicochemical parameters like chemical oxygen demand (COD) or different nitrogenous compounds to include them as part of the analysis process (69–72).

## 5 Conclusions

The findings of this study highlight the effectiveness of wastewater-based epidemiology as a viable tool for monitoring SARS-CoV-2 infection trends at the community level.

The optimized RT-qPCR method for quantifying the N1 gene of SARS-CoV-2 reported in this study has a detection limit of 10 copies/μL with a runtime of 30 to 35 min using separately available reagents. Additionally, it can be optimized for even smaller volumes or adapted to different enzyme brands if necessary. This makes it a viable alternative to commercial test kits, which can be more expensive or have limited availability.

The various sample processing methods used for detecting SARS-CoV-2, along with inherent variations in population behavior, contribute to variability in the results. Despite this, the raw data suggest that infection trends in the target population–both increases and decreases–can be tracked using this direct wastewater monitoring method integrating information on drainage systems with local population size. Even with a relatively small number of samples, this approach demonstrates effectiveness compared to traditional epidemiological monitoring methods, however, incorporating a method to normalize population size would be necessary to further improve the model.

When comparing the genome count data for the N1 gene with reported infection data, the application of computational methods for data interpolation on missing dates, along with data smoothing using the central simple moving average technique, revealed a time lag of 5 to 9 days. Additionally, a cross-correlation factor ranging from 0.825 to 0.924 was observed between the genome detection curves and reported infection curves. This lag can serve as an early warning for infections in monitored populations, allowing for the implementation of public health contingency measures if needed.

The method presented here can be replicated in other populations, provided that sampling points in the sewage system are carefully selected. Combined with a well-planned collection schedule, this approach can help validate and improve the proposed model.

Both the sample processing techniques and computational analysis methods can be further refined through continuous feedback, which would enhance the effectiveness of the proposed monitoring system.

In conclusion, we have shown how WBE offers a viable approach for monitoring SARS-CoV-2 and potentially other pathogens. Future research should integrate more variables, such as mobility patterns and climatic factors, to refine predictive models and enhance public health interventions.

## Data Availability

The datasets presented in this study can be found in online repositories. The names of the repository/repositories and accession number(s) can be found at: https://github.com/miguelatlsm/EpiCovid_MexicoCity.
