# Supplementary material for: Temporal dynamics of SARS-CoV-2 detection in wastewater and population infection trends in Mexico City
Source: Front Public Health. 2025 Aug 14;13:1640581. doi: 10.3389/fpubh.2025.1640581 (PMC12391079; doi:10.3389/fpubh.2025.1640581)
Supplement: Supplementary file 1 [file Data_Sheet_1.pdf]

Supplementary Material

1 SUPPLEMENTARY FIGURES

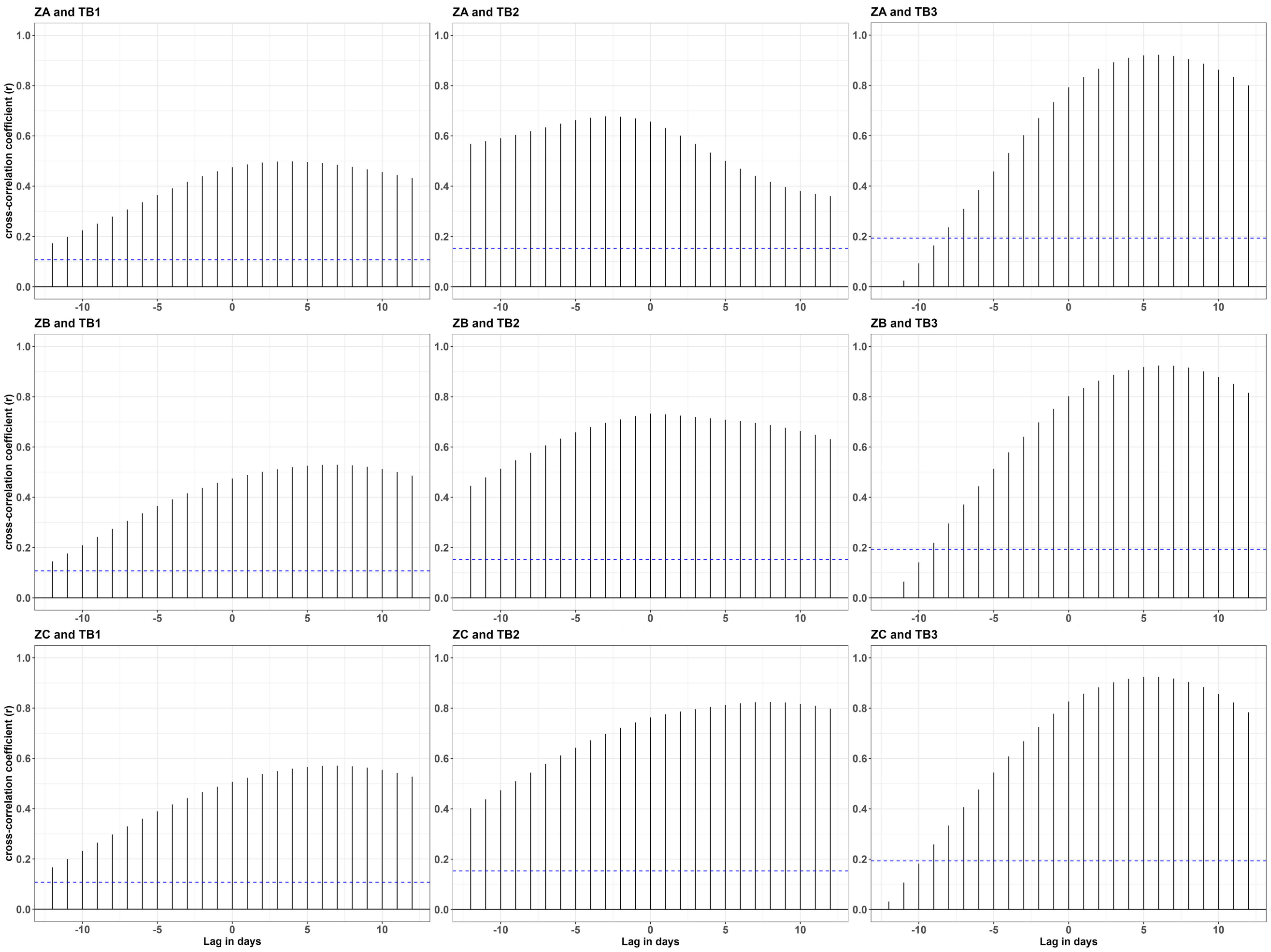

Figure S1: Graphs associated with the results of the cross-correlation factor (CCF) analysis for the combinations between the defined study areas (ZA, ZB, ZC) and the different time blocks (TB1, TB2, TB3) after the interpolation and smoothing process.

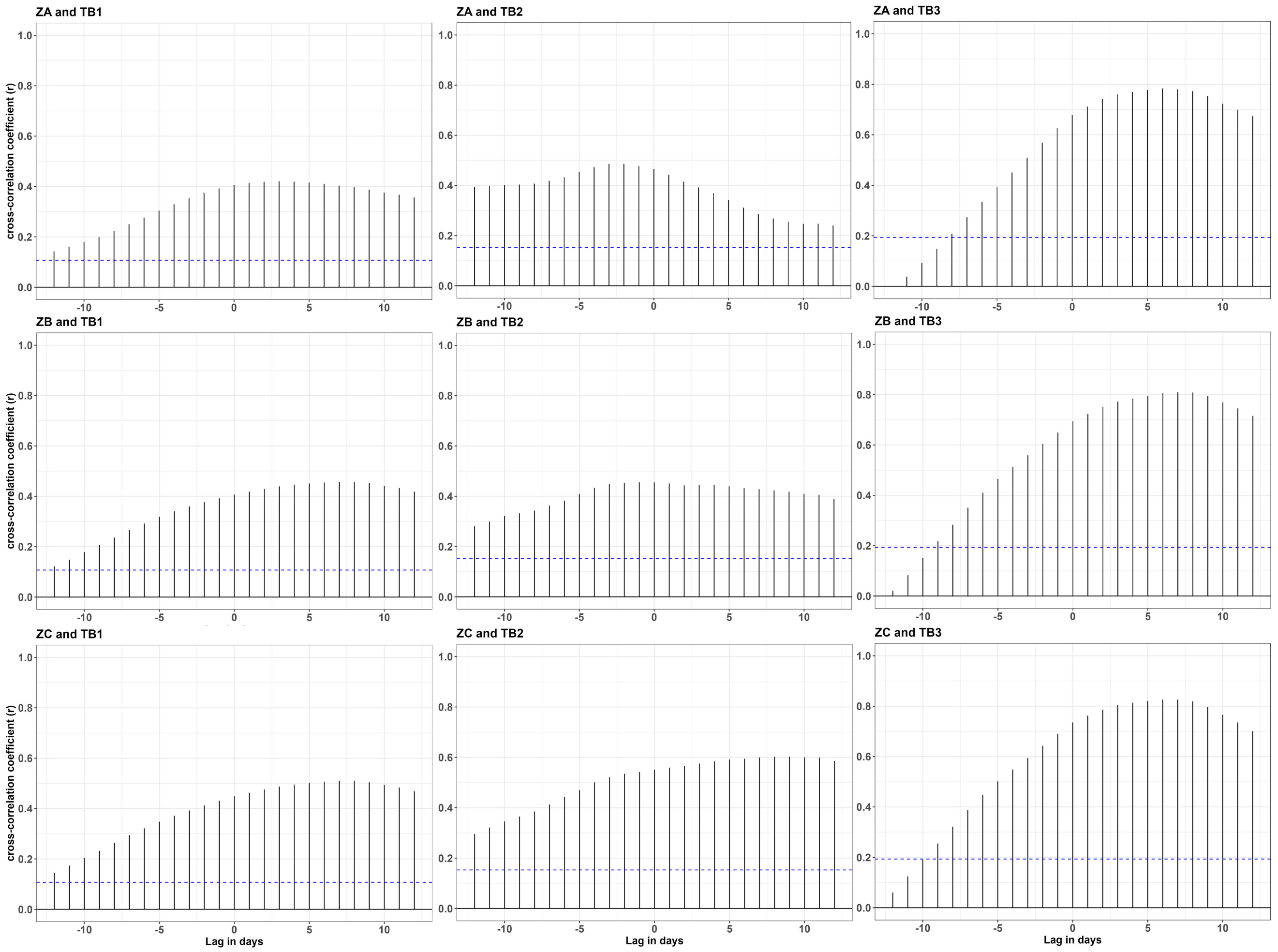

Figure S2: Graphs associated with the results of the cross-correlation factor (CCF) analysis for the combinations between the defined study areas (ZA, ZB, ZC) and the different time blocks (TB1, TB2, TB3) after the interpolation process.

	TB1			TB2			TB3		
**Zone**	**Lag (d)**	**CCF$_I$**	**CCF$_{I+S}$**	**Lag (d)**	**CCF$_I$**	**CCF$_{I+S}$**	**Lag (d)**	**CCF$_I$**	**CCF$_{I+S}$**
**Zone A**	4D	0.419	0.498	-3D	0.486	0.678	6D	0.784	0.922
	3D	0.420	0.498	-2D	0.486	0.676	5D	0.778	0.919
	5D	0.416	0.496	-4D	0.473	0.672	7D	0.780	0.917
	2D	0.418	0.494	-1D	0.477	0.669	4D	0.770	0.909
	6D	0.410	0.492	-5D	0.455	0.662	8D	0.773	0.905
	1D	0.414	0.486	0D	0.465	0.657	3D	0.760	0.892
	7D	0.403	0.485	-6D	0.433	0.649	9D	0.753	0.887
	8D	0.397	0.477	-7D	0.418	0.634	2D	0.741	0.866
	0D	0.406	0.475	1D	0.442	0.631	10D	0.723	0.863
	9D	0.386	0.468	-8D	0.401	0.622	1D	0.720	0.850
**Zone B**	3D	0.503	0.586	-4D	0.540	0.710	5D	0.779	0.897
	2D	0.500	0.582	-3D	0.542	0.708	4D	0.776	0.897
	4D	0.498	0.580	-5D	0.529	0.705	6D	0.775	0.896
	1D	0.493	0.573	-2D	0.533	0.702	3D	0.772	0.890
	5D	0.488	0.571	-6D	0.518	0.696	7D	0.769	0.888
	0D	0.489	0.570	-1D	0.522	0.693	2D	0.751	0.884
	6D	0.482	0.563	-7D	0.503	0.688	8D	0.760	0.881
	7D	0.478	0.560	-8D	0.486	0.682	9D	0.755	0.873
	8D	0.470	0.553	-9D	0.469	0.671	1D	0.751	0.864
	9D	0.465	0.548	-10D	0.452	0.667	11D	0.745	0.851
**Zone C**	7D	0.510	0.571	8D	0.602	0.825	6D	0.826	0.924
	6D	0.507	0.570	7D	0.600	0.823	5D	0.821	0.924
	8D	0.510	0.569	9D	0.603	0.823	7D	0.826	0.918
	5D	0.502	0.566	6D	0.595	0.819	4D	0.814	0.917
	9D	0.504	0.563	10D	0.600	0.818	8D	0.819	0.904
	4D	0.495	0.559	5D	0.591	0.813	3D	0.804	0.903
	10D	0.494	0.554	11D	0.599	0.810	9D	0.797	0.884
	3D	0.487	0.549	4D	0.584	0.805	2D	0.786	0.883
	11D	0.484	0.542	12D	0.586	0.798	1D	0.763	0.857
	2D	0.476	0.537	3D	0.576	0.796	10D	0.766	0.856

Table S1. Results of the cross-correlation factor (CCF) analysis for the combinations between the defined study areas (ZA, ZB, ZC) and the different time blocks (TB1, TB2, TB3), using the interpolation process (CCF$_I$) and the interpolation and smoothing process (CCF$_{I+S}$).
